# One-stage posterior laminectomy with instrumented fusion and foraminotomy for cervical ossification of posterior longitudinal ligament with radiculopathy pain

**DOI:** 10.1186/s13018-021-02431-4

**Published:** 2021-04-26

**Authors:** Bao Su, Jieliang Shen, Xiaoji Luo, Zhengxue Quan, Dianming Jiang, Xiaohua Peng, Ke Tang

**Affiliations:** 1 Department of Orthopaedics, The First Affiliated Hospital of Chongqing Medical University, 1st Youyi Road, Chongqing, 400016 People’s Republic of China; 2 Department of Orthopaedics, The Third Affiliated Hospital of Chongqing Medical University, No 1 Shuanghu Road, Chongqing, 401120 People’s Republic of China; 3 Department of Rehabilitation, The First Affiliated Hospital of Chongqing Medical University, 1st Youyi Road, Chongqing, 400016 People’s Republic of China

**Keywords:** Laminectomy, Foraminotomy, Posterior longitudinal ligament ossification, Radicular pain

## Abstract

**Objective:**

To explore the clinical efficacy of posterior LFF for cervical OPLL with radicular pain of upper limbs

**Methods:**

Between January 2014 and January 2018, 48 OPLL patients with radicular pain symptoms of upper limbs who underwent a one-stage posterior laminectomy and instrumented fusion with/without foraminotomy were reviewed retrospectively and divided into two groups: LF group (laminectomy with instrumented fusion without foraminotomy) and LFF group (laminectomy with instrumented fusion and foraminotomy). Clinical data were assessed and compared between the two groups. The radicular pain of upper limbs and neck was measured using the visual analog scale (VAS). The neurological function was evaluated with the American Spinal Injury Association (ASIA) scale. Changes of sagittal alignment were investigated by postoperative plain x-ray or computed tomography (CT). Moreover, the decompression of the spinal cord was evaluated based on postoperative MRI.

**Results:**

All the 48 patients were followed up for 24–42 months with an average follow-up time of 31.1±5.3 months. A total of 56 cervical intervertebral foramens were enlarged in 48 patients, including 40 cases (83.3%) with 1 intervertebral foramen enlargement and 8 cases (16.7%) with 2 intervertebral foramen enlargements. There were no significant differences in intraoperative blood loss, postoperative drainage amount, Japanese Orthopaedic Association (JOA) scores, JOA recovery rates, VAS scores for neck pain, and ASIA grade between two groups. The mean operative time was shorter in the LF group compared with the LFF group. The VAS score for arm pain was significantly lower while the surgical duration was longer in group B. No statistical difference was observed between the two groups in terms of C2–C7 SVA, cervical lordosis, focal angulation at the foraminotomy segment, and local spinal cord angle. Compared with the LF group, there was no segmental kyphosis or instability where the additional posterior foraminotomy was performed in the LFF group.

**Conclusions:**

One-stage posterior LFF can achieve satisfied clinical efficacy in improving neurological function and relieving the radicular pain of the upper limbs for OPLL patients with radiculopathy symptoms.

## Introduction

Ossification of the posterior longitudinal ligament (OPLL) is a multifactorial disease manifesting as an ectopic bone formation of the posterior longitudinal ligament that causes reduced range of cervical motion and spinal cord compression [1, 2]. Surgical treatment should be considered for patients with neurologic symptoms such as myelopathy and/or radiculopathy with definite evidence of neurological impairment. A variety of surgical approaches have been reported with unique risks and benefits [3–7]. However, the posterior approach is more suitable for severe canal stenosis or longer segments (≥ 3 levels) of spinal cord compression with K-line positive [8, 9].

In clinic practice, most OPLL patients suffer both myelopathy symptoms and radiculopathy pain of the upper limbs [10]. For these patients, traditional laminectomy with instrumented fusion (LF) cannot achieve effective decompression of nerve root, or even aggravate the radiculopathy symptoms and tension of nerve root due to the backward drift of the spinal cord. Kudo et al. [11] reported a case of successful foraminotomy for severe bilateral C5 palsy following posterior decompression and fusion surgery for cervical OPLL. We performed the posterior laminectomy with instrumented fusion and foraminotomy (LFF), which can simultaneously achieve the decompression of the spinal cord and the corresponding nerve root. The purpose of our study is to compare retrospectively the clinical and imaging outcomes using LFF and LF for OPLL patients with myelopathy symptoms and radiculopathy pain.

## Materials and methods

### Patient population

This is a retrospective cohort study performed in authors’ spinal care center between January 2014 and January 2018 involving 48 consecutive OPLL patients (28 men and 20 women) with myelopathy symptoms and radiculopathy pain who underwent the posterior approach operation. These patients were enrolled in the study and divided into LF group and LFF group. The study protocol conformed to the ethical guidelines of the Declaration of Helsinki and the Ethics Committee of The First Affiliated Hospital of Chongqing Medical University, Chongqing, China, and all patients provided informed consent concerning the use of their medical records. In addition, the study has been reported in line with the STROCSS criteria [12]. Preoperative diagnosis was based on history, X-rays, computed tomography (CT), and magnetic resonance imaging (MRI). Indications for surgery were neurological symptoms due to myelopathy and radiculopathy. The inclusion criteria were as follows: (1) patients were followed-up more than 24 months, (2) patients suffered myelopathy and radiculopathy due to foraminal stenosis according to MRI or CT, (3) patients were treated by LF or LFF for decompression, (4) the K-line was negative. The exclusion criteria were as follows: (1) patients were diagnosed as cervical deformity, cervical intervertebral disc herniation, multilevel cervical spondylotic myelopathy, cervical infection, or fracture; (2) patients suffered myelopathy without radiculopathy; (3) patients were followed-up for less than 24 months; (4) patients suffered cervical segmental instability or kyphosis.

### Surgical technique

**Fig. 1:**
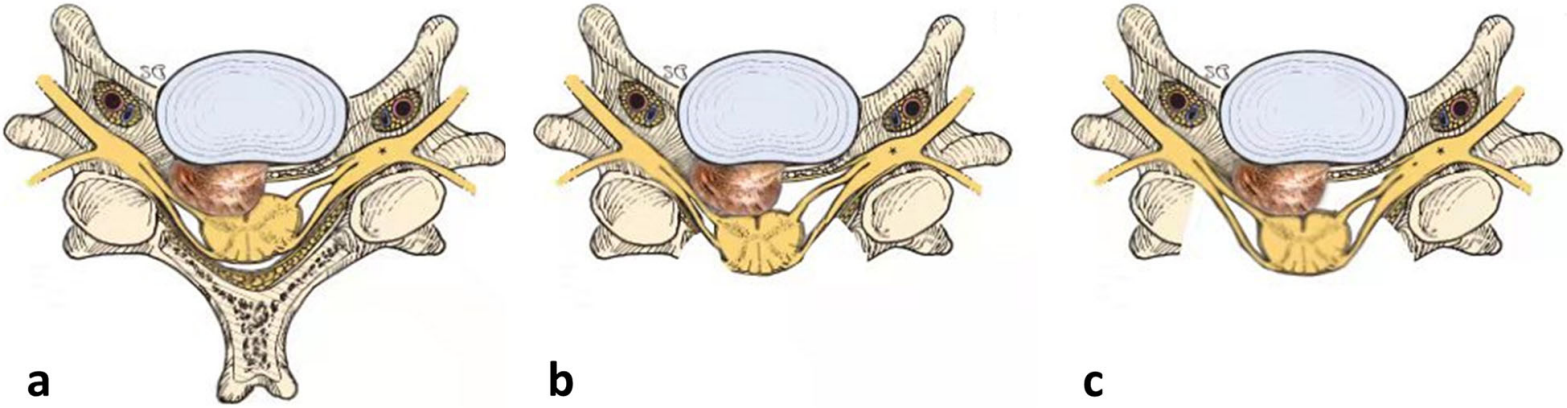
Surgical procedures of LFF. **a** The cervical spine cord and right nerve root were compressed due to the ossification of posterior longitudinal ligament. **b** Laminectomy was performed, and the cervical spine cord was partly relieved. But the right nerve root was still compressed due to the cervical ossified posterior longitudinal ligament. **c** Additional foraminotomies at the symptomatic levels were performed to relieve the nerve root. Part (less than 50%) of the facet joint was removed

All the surgeries were performed by the same senior spine surgeon in prone posture. In LF group, a laminectomy with bilateral lateral mass or pedicle screws (Stryker Spine, Cestus, France) was performed via a midline vertical incision. The decompression levels were determined according to the spinal cord compression on MRI. In the LFF group, after the LF procedures, additional foraminotomies at the symptomatic levels were performed to relieve the nerve roots. Part of the inferior articular process of the vertebra above and the superior articular process of the vertebra below were removed with piezosurgery and laminectomy rongeur. While removing the posterior wall of the intervertebral foramen, the resection of the facet joint should be less than 50% to avoid segmental kyphosis and instability. The removed spinous process and the lamina were clipped into small bone blocks and implanted at involved facet joints for fusion. After the surgery, each patient was asked to use a soft collar or Philadelphia collar for 3 months. The operation procedures are shown in Fig. 1a–c.

### Clinical outcomes

The following data were used for assessment and comparison: (1) operative time, intraoperative blood loss, postoperative drainage amount, occurrence of C5 palsy and axial symptom; (2) visual analog scale (VAS) mark for arm and neck pain; (3) the American Spinal Injury Association (ASIA) grade for neurological status; (4) function evaluations including the Japanese Orthopedic Association (JOA) score and JOA recovery rate (Hirabayashi method) [13]. These outcome scores were evaluated at baseline, 3 months, 6 months, 12 months, and 2-year follow-up.

### Radiological assessment

The following radiographic indexes were used for assessment on the cervical anteroposterior or dynamic position X-rays. (1) C2–C7 sagittal vertical axis (SVA) distance, the distance between the C2 plumb line and the posterosuperior corner of the C7 vertebral body; (2) C2–C7 cervical lordosis, the angle formed by two lines between the inferior endplate of C2 and the inferior endplate of C7 on lateral cervical X-rays; (3) focal angulation of foraminotomy segment, as described previously [14] and measured by two lines drawn between the inferior endplate of the upper vertebral body and the superior endplate of the lower vertebral body in dynamic X-ray radiographs where a foraminotomy had been performed. The difference between the maximum flexion and extension was considered to reflect the angle. (4) Local spinal cord angle, defined as the angle formed by two lines tangential to the bilateral rims of the compressed spinal cord area at the maximal compression level on sagittal view of T2-weighted MRI [15].

Statistical analysis of the parameters was performed using the *t* test with the IBM SPSS software package, version 26.0 (IBM Corp., Armonk, USA). Comparisons between preoperative and postoperative parameters within the same group were made using the paired *t* test. The gender, ASIA grade, C5 palsy, and axial symptom were analyzed by Chi-squared test. A *p*-value <0.05 was deemed as statistically significant.

## Results

### Clinical outcomes

No significant difference of demographic data was found between the two groups. All the patients were followed up for at least 24 months (range 24–42months), with a mean follow-up duration of 31.3 ± 5.0 months in the LF group and 30.9± 5.6 months in the LFF group (*p*=0.42). Comparisons of clinical characteristics between the two groups are summarized in Table 1. There were no significant differences in the improvements for intraoperative blood loss, postoperative drainage amount, pre-operative and postoperative JOA scores, JOA recovery rates, and VAS scores for neck pain between groups. The VAS score for arm pain decreased from 5.9±1.7 to 3.5±1.2 in the LF group and from 5.6 ±1.9 to 2.0±1.0 in the LFF group. There was significant improvement in the LFF group compared with the LF group (*p*=0.001). The mean operative time was shorter in the LF group compared with the LFF group [98.9 ± 15.3 min and 112.5 ±12.0 min respectively, *p* =0.001] (shown in Table 1). Postoperative C5 palsy and axial symptom did not occur in the LFF group but was observed in 2 of the 24 patients (8.3%) in the LF group. The palsy and axial symptom recovered after 5 months and 2 months, respectively. Complications including major vessel injury, dural tear, and spine cord and nerve root injury were not observed in any patient in both groups.

**Table 1:** Comparisons of clinical results between the two groups

Clinical features	Group A (*n*=24)	Group B (*n*=24)	*p* value
Age (years), mean (SD)	61.0±12.6	60.5±10.1	0.89
VAS score for neck			
Preoperative	4.0±1.6	3.7±1.3	0.62
Postoperative	1.6±0.9	1.6± 1.1	0.89
2-year follow-up	0.8±0.6	0.9 ± 0.7	0.66
VAS score for arm			
Preoperative	5.9±1.7	5.6±1.9	0.632
Postoperative	3.5±1.2	2.0±1.0	0.0
2-year follow-up	1.5±0.7	0.7± 0.6	0.0
Follow-up duration (mon)	31.3 ± 5.0	30.9 ±5.6	0.81
Operative duration (min)	98.9 ± 15.3	112.5 ± 12.0	0.001
Operation blood loss (ml)	176.3± 53.4	188.1±79.4	0.55
Postoperative drainage amount (ml)	298.8± 86.6	275.4± 71.4	0.31
JOA			
Preoperative	9.3± 2.1	9.7± 2.0	0.58
Postoperative	12.7 ± 2.1	13.6 ± 1.9	0.11
JOA recovery rates	43.5% ± 22.3%	54.3% ± 19.0%	0.76

At the final follow-up, ASIA score of each patient had improved by at least one grade. The preoperative ASIA grade of the LF group was changed from grade C to grade D in 6 cases and from grade D to grade E in 18 cases. In the LFF group, ASIA grade changed from grade C to grade D in 4 cases, from grade C to grade E in 4 cases, and from grade D to grade E in 16 cases. Mann-Whitney *U* test showed no significant difference between the two groups in the ASIA grade at the end of follow-up (shown in Table 2).

**Table 2:** Comparison of neurological status between the two groups

ASIA scale	Preoperative		Postoperative	
	Group A (*n*=24)	Group B (*n*=24)	Group A (*n*=24)	Group B (*n*=24)
A	0	0	0	0
B	0	0	0	0
C	6	8	0	0
D	18	16	6	4
E	0	0	18	20

### Radiological changes

**Fig. 2:**
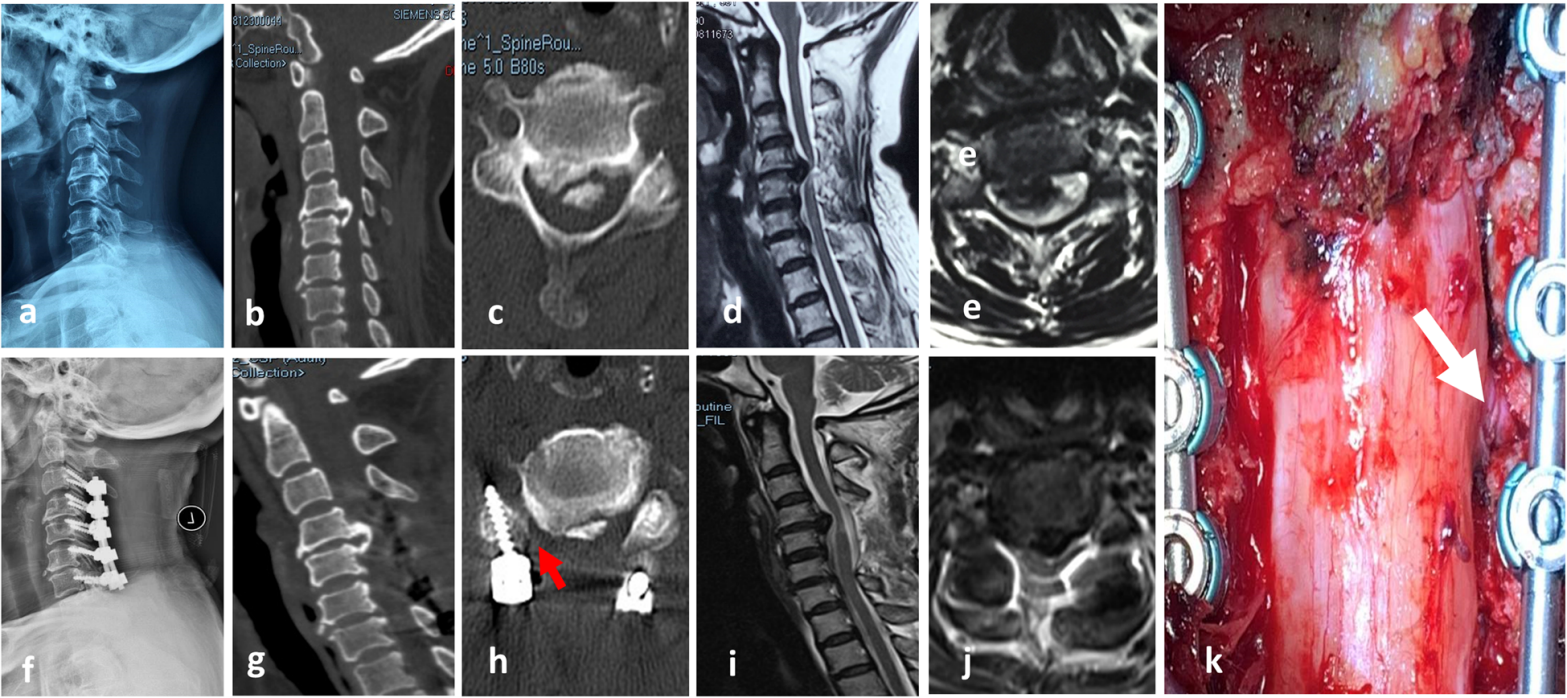
Laminectomy with instrumented fusion with foraminotomy group (LFF group). A 53-year-old female patient with progressive myelopathy and right C5 radiculopathy due to severe cervical OPLL (**a**–**e**). Preoperative X-ray, CT, and MRI images showed OPLL from C4 to C5, continuity between the rostral ossification regions and lordotic alignment of the cervical spine. **f**, **g** Postoperative X-ray and CT showed laminectomy was performed between C3 and C6 segment with maintenance of cervical lordosis. **h** Postoperative CT axial images showed the cutting margin and right posterior foraminotomy at C4/5 (red arrow). **i**, **j** Postoperative MR sagittal image showed good decompression of the spinal cord. **k** Right foraminotomy at C4/5 was performed, and the right C5 nerve root was relieved completely (white arrow)

**Fig. 3:**
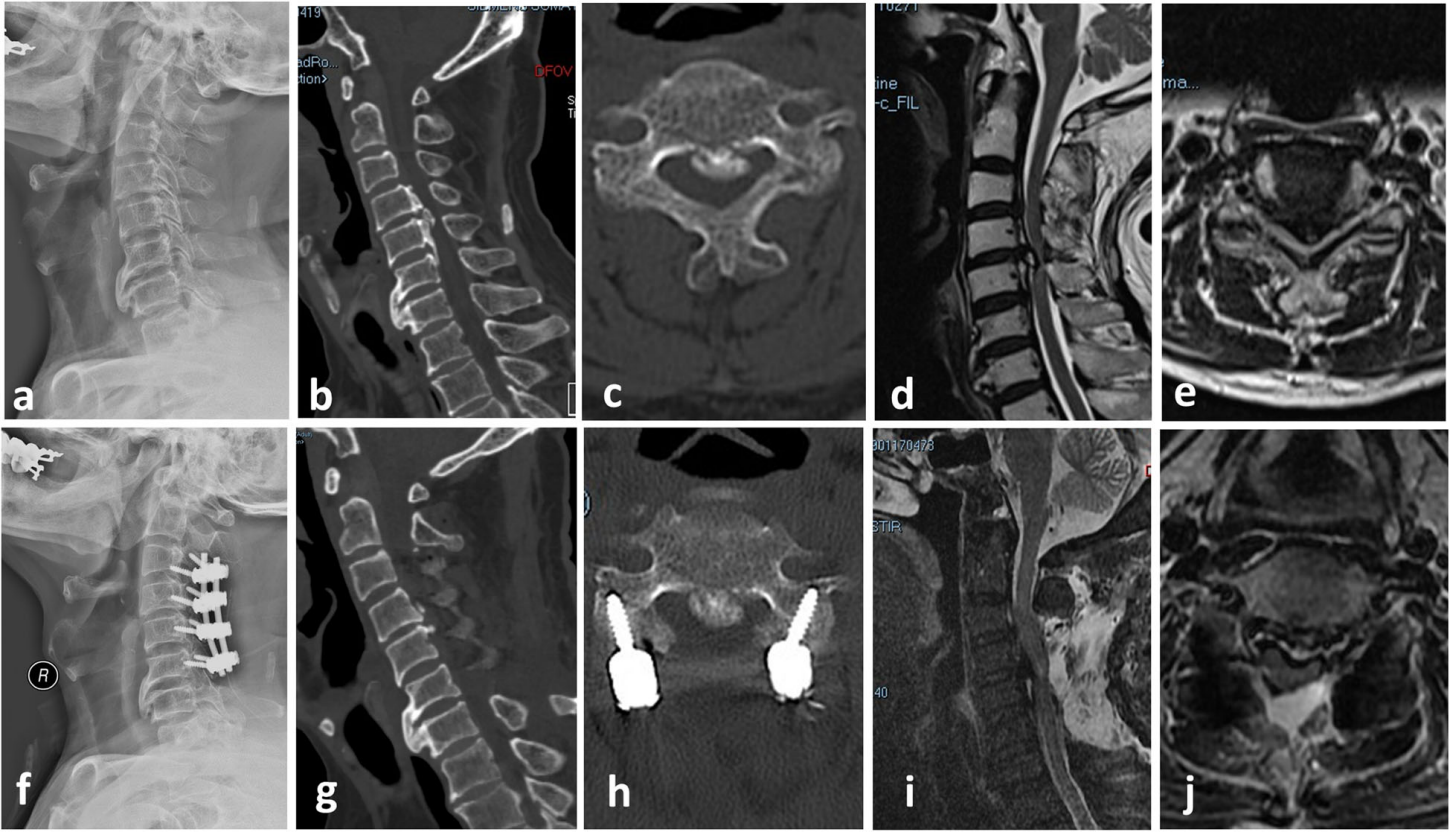
Laminectomy with instrumented fusion (LF group). A 78-year-old male patient with progressive myelopathy and right C5 radiculopathy due to severe cervical OPLL (**a**–**e**). Preoperative X-ray, CT, and MRI images showed OPLL from C4 to C6, discontinuity between the rostral ossification regions and lordotic alignment of the cervical spine. **f** Postoperative X-ray showed laminectomy was performed between C3 and C6 segment with maintenance of cervical lordosis. **g**–**j** Postoperative CT and MR sagittal image showed decompression of the spinal cord

A summary of the radiographic changes is provided in Table 3. No statistical difference was observed between the two groups before surgery in terms of C2–C7 SVA, cervical lordosis, focal angulation at the foraminotomy segment, and local spinal cord angle before surgery (*p* > 0.05). Preoperative, immediately postoperative, and 1-year follow-up dynamic lateral radiographs in the LF group showed that although C2–C7 SVA increased and cervical lordosis decreased postoperatively in both groups, there were no significant differences in the degree of reduction between the LF and LFF groups (*p*=0.759 and 0.828, respectively). The focal angulation had changed from 6.3°±2.9° to 4.8°±2.5° in the LF group and from 6.0°±2.7° to 4.5°±2.4° in the LFF group. The local spinal cord angle changed from 31.5°±7.8° to 20.5°±4.2° in the LF group and from 30.8°±7.7° to 21.0°±6.7° in the LFF group. However, postoperative focal angulation and local spinal cord angle had not significantly changed in either group (*p*=0.62 and 0.78, respectively). Compared with the LF group, there was no segmental kyphosis or instability where the additional posterior foraminotomy was performed in the LFF group. Representative radiological images of one patient in each group are shown in Figs. 2 and 3. Comparisons of radiological changes between the two groups are summarized in Table 3.

**Table 3:** Comparison of radiological changes between the two groups

Clinical features	Group A (*n*=24)	Group B (*n*=24)	*p* value
SVA (mm)			
Preoperative	24.9±7.6	26.0±9.2	0.63
Immediately postoperative	29.5±9.5	31.1±10.9	0.57
1-year follow-up	29.1±9.5	30.9±10.0	0.53
Cervical lordosis (°)			
Preoperative	15.5±7.1	13.4±6.6	0.30
Immediately postoperative	13.9±6.3	11.2±5.8	0.13
1-year follow-up	12.3±5.8	9.7±5.8	0.13
Focal angulation (°)			
Preoperative	6.3±2.9	6.0±2.7	0.70
Immediately postoperative	5.2±2.5	5.7±2.6	0.68
1-year follow-up	4.8±2.5	4.5±2.4	0.62
Local spinal cord angle (°)			
Preoperative	31.5±7.8	30.8±7.7	0.75
Immediately postoperative	21.1±4.0	20.5±6.8	0.74
1-year follow-up	20.5±4.2	21.0±6.7	0.78

## Discussion

The optimal method for multilevel OPLL remains controversial [16]. Anterior decompression and direct removal of the ossified posterior longitudinal ligament seems to be radical since the major pathomechanism of OPLL is anterior neurological compression. However, anterior approach becomes more technically demanding and has higher risk of complications such as spinal cord injury, dural tears, or hemorrhoea, with the increasing narrowing extent of ossification [5]. Therefore, posterior decompression is the preferred choice of surgical treatment for multilevel cervical OPLL due to its relatively safer procedure for severe canal stenosis or 3 or more levels of OPLL.

Laminoplasty (LP) and LF are considered as reliable and effective posterior approach, of which LF can increase spinal canal volume more effectively and has lower risk for kyphotic and OPLL progression than that with LP [17]. However, LF may result in the backward shifting of the spinal cord which leads to the stretching of the nerve root. Therefore, it is difficult to relieve the radiculopathy only by LF approach. Up to our best knowledge, this study is the first time to compare the clinical and radiological outcomes between LF and LFF for treating cervical OPLL with radicular pain of upper limbs. However, posterior laminectomy with foraminotomy was used in some researches to treat cervical radiculo-myelopathy. In a study by Fang et al., the effectiveness of posterior laminectomy and lateral mass screw fixation combined with foraminotomy for treating cervical radiculo-myelopathy (not for OPLL) was evaluated [18]. In this study, the mean operation time was longer than ours (mean 204 min, range from 167 to 260 min), and a total of 135 foramens were dissected, with an average of 2.33 foramens per patient. They used high-speed burr for laminectomy, while in our research, we used piezosurgery. And only an average of 1.4 cervical intervertebral foramens was enlarged for one patient in our research. Due to the usage of piezosurgery and less foraminotomy for one patient, the duration of time of our surgery was shorter. What’s more, the JOA recovery rates were 68.5%± 21.9% in their research compared with 54.3% ± 19.0% in our research. This may be related to the progression of OPLL.

Our results demonstrated that the VAS for arm pain decreased more in LFF group than that in LF group, which evaluated radiculopathy caused by nerve root compression at the intervertebral foramina. For the potential mechanism, foraminal stenosis at the anterolateral corner of the spinal canal will put more compression on the corresponding nerve roots, which is one of the most common clinical courses of cervical radiculopathy. Tanaka et al. [19] reported that the intervertebral foramina are shaped like a funnel, where the entrance zone (medial half of the intervertebral foramen) was the narrowest part and exit zone (lateral half of the intervertebral foramen) was the widest. Therefore, when the OPLL occurs, compression of the nerve roots often occurred at the entrance zone of the intervertebral foramina. In posterior cervical foraminotomy, the nerve roots can be decompressed by resecting the medial half of the facet joints.

Foraminotomy is usually performed for one- or two-level unilateral upper extremity radiculopathy due to posterolateral or foraminal disk herniation or disk/osteophyte complex [20]. Theoretically, cervical instability would occur after the resection of facet joint following foraminotomy. However, this study showed no significant trend toward cervical kyphosis, angulation, or slippage in LFF group compared with the LF group. The possible reasons are as follows: (1) Zdeblick et al. found segmental hypermobility of the cervical spine that occurs if a foraminotomy involves resection of more than 50% of the facet [21]. In this study, the posterior wall of intervertebral foramen was removed less than 50% to ensure the stability of the cervical spine for the patients in the LFF group. (2) Posterior fixation with local bone graft fusion was performed for each patient. This may provide stronger biomechanical strength and reduce post-laminectomy kyphosis and instability. Hence, we suggest that LFF could achieve good surgical results for OPLL with radiculopathy without segmental kyphosis or instability.

C5 nerve palsy is one of the most common complications after posterior cervical surgery [22]. The intervertebral foramen tends to be narrower at C4 and C5 while the ossified posterior longitudinal ligament is often the thickest at the C5 segment [23]. In addition, a high incidence of anterior osteophyte formation of the superior facet joint and the posterior ramus proper is shortest at the C4 and C5 nerves [24]. These features might lead to a “tethering effect” that stretches and compresses the C5 nerve root at the intervertebral foramen and radiculopathy pain. The lower occurrence of C5 nerve palsy in the LFF group may be attributed to the foraminotomy of the C4-C5.

There are few limitations that are associated with this study. First, the retrospective nature of this study did not allow us to prospectively test our hypothesis. Moreover, the case series was limited based on a single center’s experience. The follow-up period was relatively short (mean 31.1 months). Future studies including prospective, randomized study with longer follow-up period will be required to determine the durability of these findings.

In conclusion, LFF can provide satisfactory results in OPLL patients with myelopathy and radicular symptoms of upper limbs. LFF method can achieve satisfied clinical efficacy in improving neurological proper cases.

## Data Availability

The data used to support the findings of this study are available from the corresponding author upon request.

## Abbreviations

ASIA: American Spinal Injury Association
CRP: C-reactive protein
CT: Computed tomography
ESR: Erythrocyte sedimentation rate
LIF: Laminectomy with instrumented fusion
LIFF: Laminectomy with instrumented fusion and foraminotomy
MRI: Magnetic resonance imaging
OPLL: Ossification of the posterior longitudinal ligament
VAS: Visual analog scale
